# Identifying critical modules and biomarkers of intervertebral disc degeneration by using weighted gene co‐expression network

**DOI:** 10.1002/jsp2.70004

**Published:** 2024-10-18

**Authors:** Daqian Zhou, Tao Liu, Yongliang Mei, Jiale Lv, Kang Cheng, Weiye Cai, Silong Gao, Daru Guo, Xianping Xie, Zongchao Liu

**Affiliations:** ^1^ Department of Orthopedics, The Affiliated Traditional Chinese Medicine Hospital Southwest Medical University Luzhou Sichuan Province China; ^2^ Department of Orthopedics Luzhou Longmatan District People's Hospital Luzhou Sichuan China

**Keywords:** biomarkers, DEGs, gene expression omnibus, intervertebral disc degeneration, WGCNA

## Abstract

**Background:**

Intervertebral disc degeneration (IVDD) is an age‐related orthopedic degenerative disease characterized by recurrent episodes of lower back pain, the pathogenesis of which is not fully understood. This study aimed to identify key biomarkers of IVDD and its causes.

**Methods:**

We acquired three gene expression profiles from the Gene Expression Omnibus (GEO) database, GSE56081, GSE124272, and GSE153761, and used limma fast differential analysis to identify differentially expressed genes (DEGs) between normal and IVDD samples after removing batch effects. We applied weighted gene co‐expression network (WGCNA) to identify the key modular genes in GSE124272 and intersected these with DEGs. Next, A protein–protein interaction network (PPI) was constructed, and Cytoscape was used to identify the Top 10 hub genes. Functional enrichment analyses were performed using gene ontology (GO) and Kyoto Encyclopedia of Genes and Genomes (KEGG) databases. Three key genes were validated using Western Blot (WB) and qRT‐PCR. Additionally, we predicted miRNAs involved in hub gene co‐regulation and analyzed miRNA microarray data from GSE116726 to identify four differentially expressed miRNAs.

**Results:**

We identified 10 hub genes using bioinformatics analysis, gene function enrichment analysis revealed that they were primarily enriched in pathways, such as the TNF signaling pathway. We chose JUNB, SOCS3, and CEBPB as hub genes and used WB and qRT‐PCR to confirm their expression. All three genes were overexpressed in the IVDD model group compared to the control group. Furthermore, we identified four miRNAs involved in the co‐regulation of the hub genes using miRNet prediction: mir‐191‐5p, mir‐20a‐5p, mir‐155‐5p, and mir‐124‐3p. Using limma difference analysis, we discovered that mir‐191‐5p, mir‐20a‐5p, and mir‐155‐5p were all down‐regulated and expressed in IVDD samples, but mir‐124‐3p showed no significant change.

**Conclusion:**

JUNB, SOCS3, and CEBPB were identified as key genes in IVDD, regulated by specific miRNAs, providing potential biomarkers for early diagnosis and therapeutic targets.

## Graphical abstract

Intervertebral disc degeneration (IVDD) is an age‐related orthopedic degenerative disease characterized by recurrent episodes of lower back pain, the pathogenesis of which is not fully understood. The aim of this study was to identify key biomarkers of IVDD and its causes.

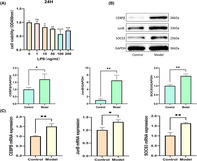

## 1 INTRODUCTION

Intervertebral disc degeneration (IVDD) is a common and incurable orthopedic degenerative condition that not only reduces people's quality of life but also causes a significant financial load on the social healthcare system.^1^ The intervertebral disc is the largest avascular tissue in the human body, which is mainly composed of three parts: the central nucleus pulposus (NP) tissue, the surrounding annulus fibrosus, and the upper and lower cartilaginous endplates.^2^ Its pathogenesis is not clear and may be related to factors such as senescence and apoptosis of nucleus pulposus cells (NPCs), inflammatory stimuli, extracellular matrix degradation (ECM), oxidative stress, and so on.^3^, ^4^, ^5^ Clinical treatment is mainly divided into conservative treatment and surgical treatment,^6^ conservative treatment is through massage, massage manipulation therapy oral non‐steroidal anti‐inflammatory drugs, and so forth, in the early stage of the disease can alleviate a certain degree of pain symptoms.^7^ Surgical treatment is to remove the protruding NP to relieve the symptoms of lower back pain, however, not all surgeries can achieve ideal results, and some patients have persistent back pain after surgery.^8^ It is clear that the clinical effectiveness and available options for IVDD are extremely limited,^9^ therefore, it has become an urgent need to clarify the pathogenesis of IVDD and to find new therapeutic targets to combat IVDD.

In recent years, rapid advances in gene microarray technology and bioinformatics analysis have given us the ability to observe the expression of thousands of genes, which has contributed to a deeper understanding of the pathophysiological role of genes in disease. High‐throughput sequencing tools have recently provided new insights into disease processes and biomarker discovery.^10^ We investigated gene expression differences (DEGs) between healthy and IVDD patient tissues using microarray datasets obtained from GEO. The application of gene‐weighted co‐expression network (WGCNA) can reveal disease‐related gene networks and co‐expressed gene modules with important biological significance,^11^ therefore, we intersected DEGs with the key modular genes obtained from the WGCNA analysis and constructed a protein–protein interaction network (PPI) to screen out the pivotal genes among them. Then, we obtained the possible molecular processes and key pathways leading to IVDD by enrichment analysis of Kyoto Encyclopedia of Genes and Genomes (KEGG) pathways and gene ontology (GO) keywords. From the 10 hub genes, we selected three hub genes, JUNB, SOCS3, and CEBPB, and evaluated them by ROC curve. In addition, we verified the expression levels of JUNB, SOCS3, and CEBPB by Western Blot (WB) and qRT‐PCR in normal and lipopolysaccharide (LPS)‐induced model groups of human NPCs. The results showed that these three genes showed a trend of overexpression in the model group, which was consistent with our expected results. Finally, we obtained the co‐regulatory miRNAs involved in the regulation of the three hub genes, JUNB, SOCS3, and CEBPB, by miRNet analysis. through the above methods, our results are expected to provide a theoretical basis for further understanding of the mechanism of IVDD and to identify potential biomarkers, which will deepen our understanding of the molecular mechanism of IVDD at the systems biology level.

## 2 MATERIALS AND METHODS

### 2.1 Microarray data source

Datasets GSE56081, GSE124272, and GSE153761 were downloaded from the Gene Expression Omnibus (GEO) database,^12^, ^13^ The microarray data for GSE56081 contains 5 IVDD samples and 5 control samples, the microarray data for GSE124272 contains 8 IVDD samples and 8 control samples, and GSE153761 contains 3 IVDD samples and 3 control samples, all of which were sourced from human subjects. The samples included five NP samples from patients with disc degeneration and five from control individuals, eight whole blood samples from patients with IVDD prolapse and eight from healthy controls, and three cervical disc endplate samples from patients with cervical myelopathy and three from patients with vertebral fracture. Dataset GSE124272 was sequenced on GPL21185, dataset GSE56081 was sequenced on GPL15314, and dataset GSE153761 was sequenced on GPL22120. To create the integrated GEO dataset, we processed the above three datasets to remove batch effects using Sangerbox 3.0.

### 2.2 Differentially expressed genes (DEGs) associated with IVDD

“Limma” is a differential expression screening method based on generalized linear models, here we used R package limma (version 3.40.6) for differential analysis to obtain differential genes between different comparator and control groups,^14^ specifically, we acquired expression profiling dataset, multiple linear regression using the lmFit function, and further used the eBays function for compute moderated *t*‐statistics, moderated *F*‐statistic, and log‐odds of differential expression by empirical Bayes moderation of the standard errors toward a common value, and finally obtain the significance of the differences for each gene.

### 2.3 Weighted gene co‐expression network analysis

We used the systems biology strategy WGCNA to explore the correlation between genes,^11^ first, the median absolute deviation (MAD) of each gene was determined, and 50% of the genes with the smallest MAD were removed. Second, the DEG expression matrix was filtered using the well‐characterized samplegenes function to eliminate the unqualified genes and samples to build the scale‐tree co‐expression network. Third, the neighbor‐joining relationship is calculated using the “soft” threshold power (β) derived from the co‐expression similarity. Neighborhoods were then transformed into topological overlap matrices to determine their gene proportions and differences. In the fourth step, the modules are detected using hierarchical clustering and dynamic tree‐cut functions. The average chain hierarchical clustering method was used to divide the genes expressing the same spectrum into multiple gene modules. Finally, the dissimilarity of the genes characterized by the modules is calculated, the cut line of the module tree is selected, and multiple modules are combined together for further study. Millions of genes are entered into modules according to their expression patterns, and each module has genes with common expression patterns. Finally, we use “Venn Diagram” to find the overlap between core module genes and DEGs.

### 2.4 PPI network analysis

Search Tool for the Retrieval of Interacting Genes (STRING) (https://cn.string-db.org/) is an online tool dedicated to analyzing functional protein association networks,^15^, ^16^, ^17^ where we intersected WGCNA core module genes and DEGs with DEGs genes in the STRING database, and genes with experimentally verified interaction composite scores >0.4 were considered significant. Subsequently, the PPI network was visualized by Cytoscape software(version 3.9.1) (www.cytoscape.org/).^18^ cytoHubba plugin in Cytoscape was used to screen the hub genes in the PPI network, where the top 10 genes were identified as hub genes.^19^

### 2.5 Functional enrichment analysis

The GO system provides structured, computable information about the function of genes and gene products,^20^ and the KEGG is a widely used database for gene system research.^21^ For gene set functional enrichment analysis we used the KEGG rest API (https://www.kegg.jp/kegg/rest/keggapi.html) to obtain up‐to‐date gene annotations for the KEGG Pathway, as well as using the R package org.Hs.eg.db (version 3.1.0) for the GO annotations of the genes were used as the background, and the genes were mapped to the background set and enriched using the R package clusterProfiler (version 3.14.3) to obtain the gene set enrichment results. The minimum gene set was set to be 5 and the maximum gene set to be 5000, *p*‐value of <0.05 and an FDR of <0.25 were considered statistically significant.

### 2.6 Experimental consumables

The Chinese Academy of Sciences' cell bank provided the immortalized human NPCs (HUM‐iCELL‐s012), which were created from intervertebral disc tissue and lentivirally transfected to carry the SV40 gene. We bought LPS from Aladdin Company. Fetal bovine serum (FBS) was obtained from Gibco Inc.; the PC‐1iCell Primary Chondrocyte Cell Culture System was acquired from HyClone Inc. with an HPLC grade of ≥94%. PC‐liCell Primary Chondrocyte Cell Culture System is used for NPCs culture and experimental validation. We bought SOCS3, CEBPB, JUNB antibody, and GAPDH antibody from Biobay Bio. Beijing Solabio Technology Co. was the supplier of the CCK‐8 kit (cell proliferation and toxicity detection assay) that was acquired.

### 2.7 Cell culture and LPS treatment

NPCs were cultured in DMEM containing 10% FBS and 1% antibiotics, maintained in a 37°C incubator with 5% CO_2_. During the experiment, cells were divided into six groups and treated with 0, 10, 20, 50, 100, and 200 ng/mL LPS for 24 h.

### 2.8 CCK8 assay

Cell viability was measured using the Cell Counting Kit‐8 (CCK8). Treated cells (100 μL) were transferred to a 96‐well plate, and 10 μL of CCK8 reagent was added to each well, followed by incubation at 37°C for 2 h. The absorbance was measured at 450 nm using a microplate reader, and cell viability was calculated.

### 2.9 Protein extraction and WB analysis

Total protein was extracted from cells using RIPA lysis buffer, and protein concentration was determined using the BCA method. Equal amounts of protein (20 μg) were separated by SDS‐PAGE and then transferred to PVDF membranes. The membranes were blocked with 5% non‐fat milk for 1 h and then incubated overnight with primary antibodies against SOCS3 (1:1000 dilution), CEBPB (1:1000 dilution), and JUNB (1:1000 dilution). The next day, the membranes were incubated with secondary antibodies (1:2000 dilution) for 1 h, and protein bands were detected using the ECL chemiluminescence method. GAPDH was used as a loading control.

#### 2.9.1 Quantitative reverse transcription PCR (qRT‐PCR)

To validate the three hub genes we identified (JUNB, SOCS3, and CEBPB), we performed in vitro experiments using qRT‐PCR. We extracted total RNA from the normal and LPS intervention model groups of myeloid cells using a total RNA extraction kit. we then reverse‐transcribed 1 μg of total RNA into cDNA using an iScript cDNA synthesis kit. to calculate the relative mRNA levels, we used the 2‐ΔΔ*Ct* technique to normalize the GAPDH and analyzed by real‐time fluorescence quantitative PCR using Bio‐Rad CFX96 equipment. Through these analyses, we obtained the relative expression of three genes, JUNB, SOCS3, and CEBPB, in the normal and model groups. Human NPCs lines were purchased from icell, Inc. (icell‐00528a). PC‐1iCell Primary Chondrocyte Cell Culture System was purchased from HyClone Inc. PS was purchased from Aladdin Company. HPLC grade (≥94%); FBS was purchased from Gibco Inc. The following is the list of primer sequences:

JUNB‐F 5′CCAGCTCAAACAGAAGGTCATGA3′

JUNB‐R 5′AAACGTCGAGGTGGAAGGACT3′

SOCS3‐F 5′CCTGCGCCTCAAGACCTTC3′

SOCS3‐R 5′GTCACTGCGCTCCAGTAGAA3′

CEBPB‐F 5′GGCCCTGAGTAATCGCTTAAAGA3′

CEBPB‐R 5′AGTGTTCTTAATGCTTGAAACGGAAA3′

GAPDH‐F 5′CCAGCAAGAGCACAAGAGGA3′

GAPDH‐R 5′TGAGGAGGGGAGATTCAGTGT3′

#### 2.9.2 Analysis of ROC curves for hub genes

To ensure the accuracy of each candidate hub gene, we used receptor operating characteristic (ROC) curves. We used the “pROC” software package for ROC curve analysis,^22^ ROC curves are a common method for assessing the performance of classification models to evaluate the accuracy and sensitivity of the model. By plotting the relationship between true positive rate and false positive rate, we can determine the performance of the model under different thresholds. In our study, we used ROC curves to evaluate the accuracy of each candidate hub gene in disease diagnosis. The “pROC” software package was used for ROC curve analysis because it provides a rich set of features and tools to easily calculate the area under curve (AUC), which is the area under the ROC curve and is used to measure the performance of a classifier. In general, the larger the AUC value, the better the performance of the classifier. In our study, we considered genes with an area under the curve greater than 0.70 as possibly having diagnostic value for the disease. This threshold was chosen based on experimental experience and previous studies and may vary depending on the characteristics of the specific disease and study population. By selecting genes with high AUC values as hub genes, we can determine the importance of these genes in the diagnosis of the disease, as well as provide a strong basis for subsequent studies.

#### 2.9.3 Prediction of target miRNAs involved in hub gene co‐regulation

In order to perform target miRNA prediction involved in the co‐regulation of hub genes, in this study, we used the miRNet tool (www.mirnet.ca/),^23^ In using the miRNet tool to predict miRNA targets, we first input the list of target genes into miRNet and select the appropriate parameter settings for analysis. miRNet will predict the regulatory miRNAs associated with our target genes by integrating the information from multiple miRNA databases and applying the raw confidence algorithm and prediction model. this process helps us to understand the interaction network between hub genes and miRNAs.

To validate the differential expression of miRNAs predicted by miRNet, we selected relevant miRNA microarray data from the GEO database (GSE116726). We processed these data using the Limma rapid differential analysis method to identify significantly differentially expressed miRNAs consistent with our research objectives. The Limma analysis method identifies DEGs by using linear models and statistical tests in between‐group comparisons.

This integrated miRNet and Limma approach provides accurate miRNA prediction results and filters out miRNAs that are functionally important and differentially expressed in the regulation of hub genes. through this approach, we were able to gain insights into the regulatory relationship between hub genes and miRNAs, which is important for further investigation of the biological mechanisms and identification of potential biomarkers.

### 2.10 Statistical analysis

Our study data were statistically analyzed using GraphPad Prism 9.3. The *t*‐test and Kruskal–Wallis were used to test whether the data were statistically significant. Where ns indicates no significance; * indicates *p* < 0.05; ** indicates *p* < 0.01; *** indicates *p* < 0.001; and **** indicates *p* < 0.0001.

## 3 RESULTS

### 3.1 Identification of DEGs

**FIGURE 1:**
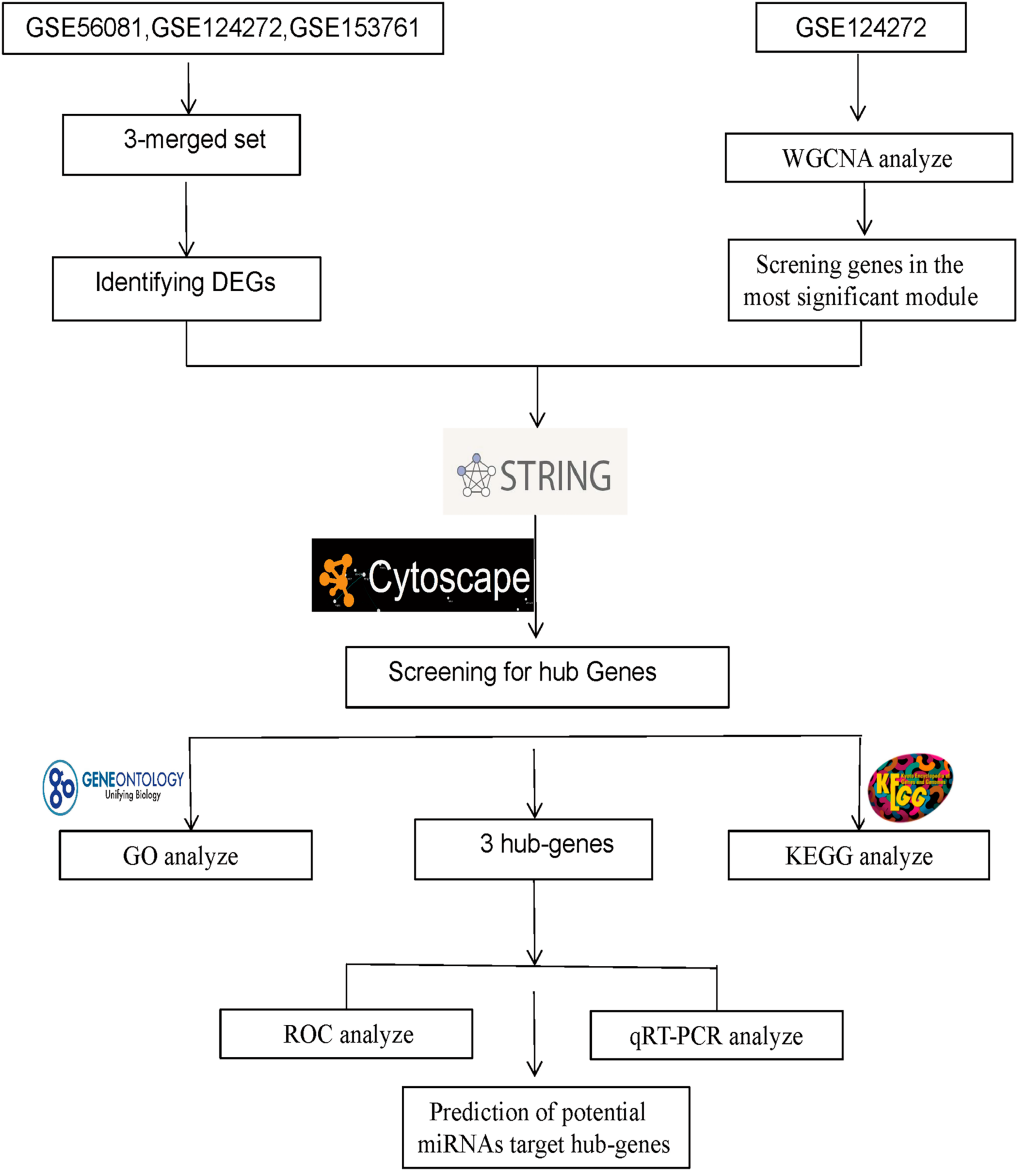
Flow chart of this study.

**FIGURE 2:**
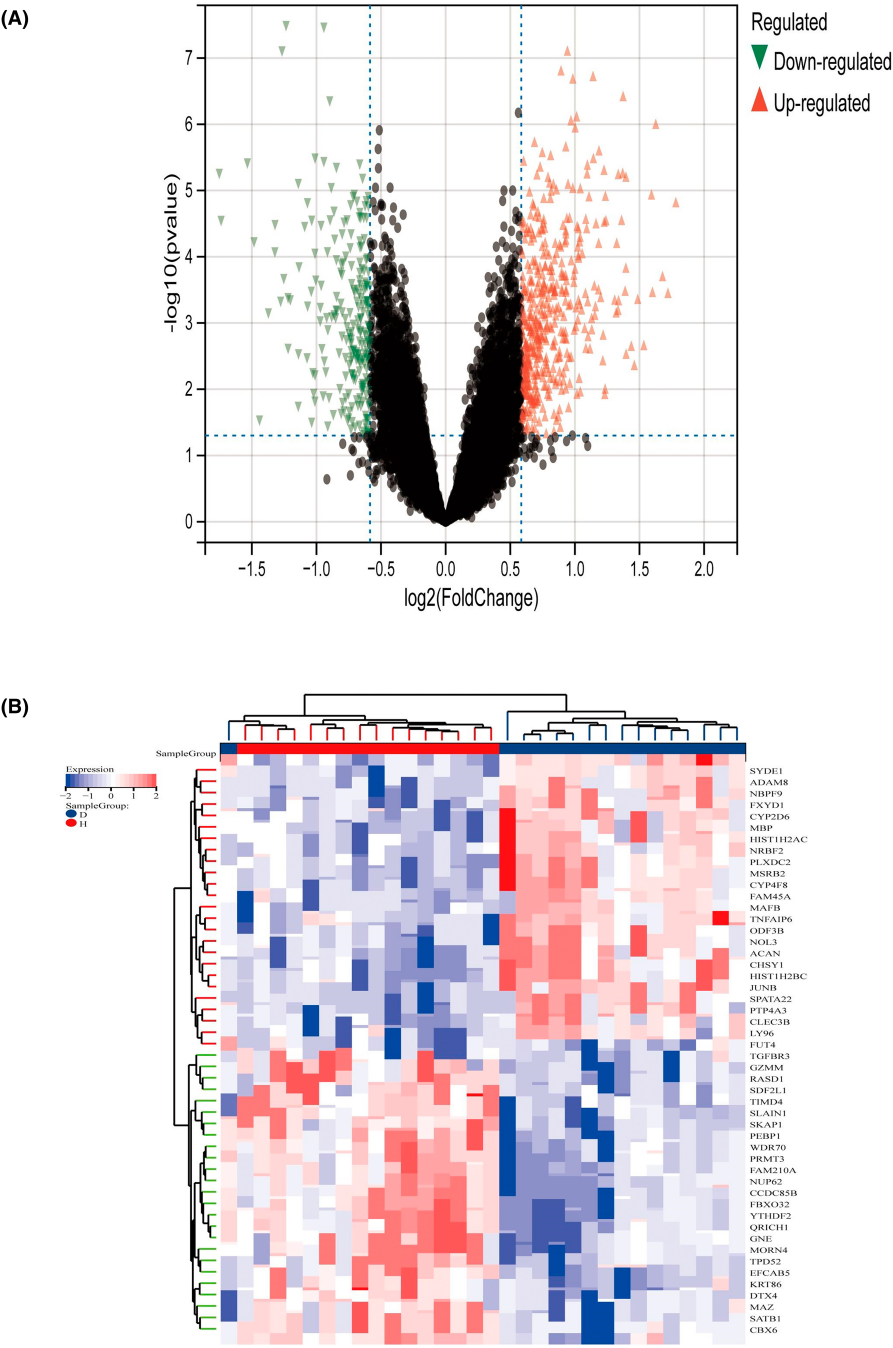
Heatmap and volcano plot for the DEGs identified from the integrated IVDD dataset. (A) Red and green plot triangles represent DEGs with upregulated and downregulated gene expression. (B) Each row displays DEGs and each column refers to one of the IVDD case or control group samples. Red and blue colors represent DEGs with up‐ and down‐regulated gene expression. H, normal control group; D, IVDD disease group. *p* <0.05 和 |log2FC| >1.

Figure 1 demonstrates our research process, in order to identify the DEGs, we first selected GSE56081, GSE124272, and GSE153761 from the GEO dataset as our research subjects. We processed these datasets to remove the batch effect to ensure the reliability of the results from the acquisition of the datasets to the final identification of the DEGs. To obtain an integrated dataset, we used the Limma rapid differential analysis method. In this step, we successfully identified 496 DEGs. Among them, the expression of 329 genes was up‐regulated, while 167 genes were down‐regulated. We used heatmaps and volcano plots for visualization and analysis to highlight the pattern of DEGs in a more accessible way. (Figure 2) depicts heatmaps and volcano plots of IVDD DEGs, illustrating gene expression patterns and the significance of discrepancies. The genes that are upregulated in the IVDD group, such as JUNB, SYDE1, ADAM8, and MAFB, are primarily involved in inflammatory responses, extracellular matrix remodeling, and cellular stress responses, which are processes known to be associated with intervertebral disc degeneration. For instance, JUNB is a component of the AP‐1 transcription factor complex and plays a role in regulating gene expression in response to inflammatory signals. Similarly, ADAM8 is involved in proteolysis and cell adhesion, contributing to the degradation of the extracellular matrix.

Conversely, the genes that are downregulated in the IVDD group, including TGFBR3, FUT4, and SATB1, are associated with cellular signaling, immune modulation, and chromatin organization. TGFBR3 is part of the TGF‐β receptor complex, which plays a critical role in maintaining tissue homeostasis and regulating immune responses. The downregulation of these genes may reflect impaired signaling pathways and altered cellular functions in the degenerative disc environment.

We may further examine the activities and regulatory networks of these genes by identifying and analyzing the DEGs, revealing their key involvement in IVDD. These findings give substantial support and direction for our further study.

### 3.2 Identification and analysis of key module of IVDD by WGCNA

**FIGURE 3:**
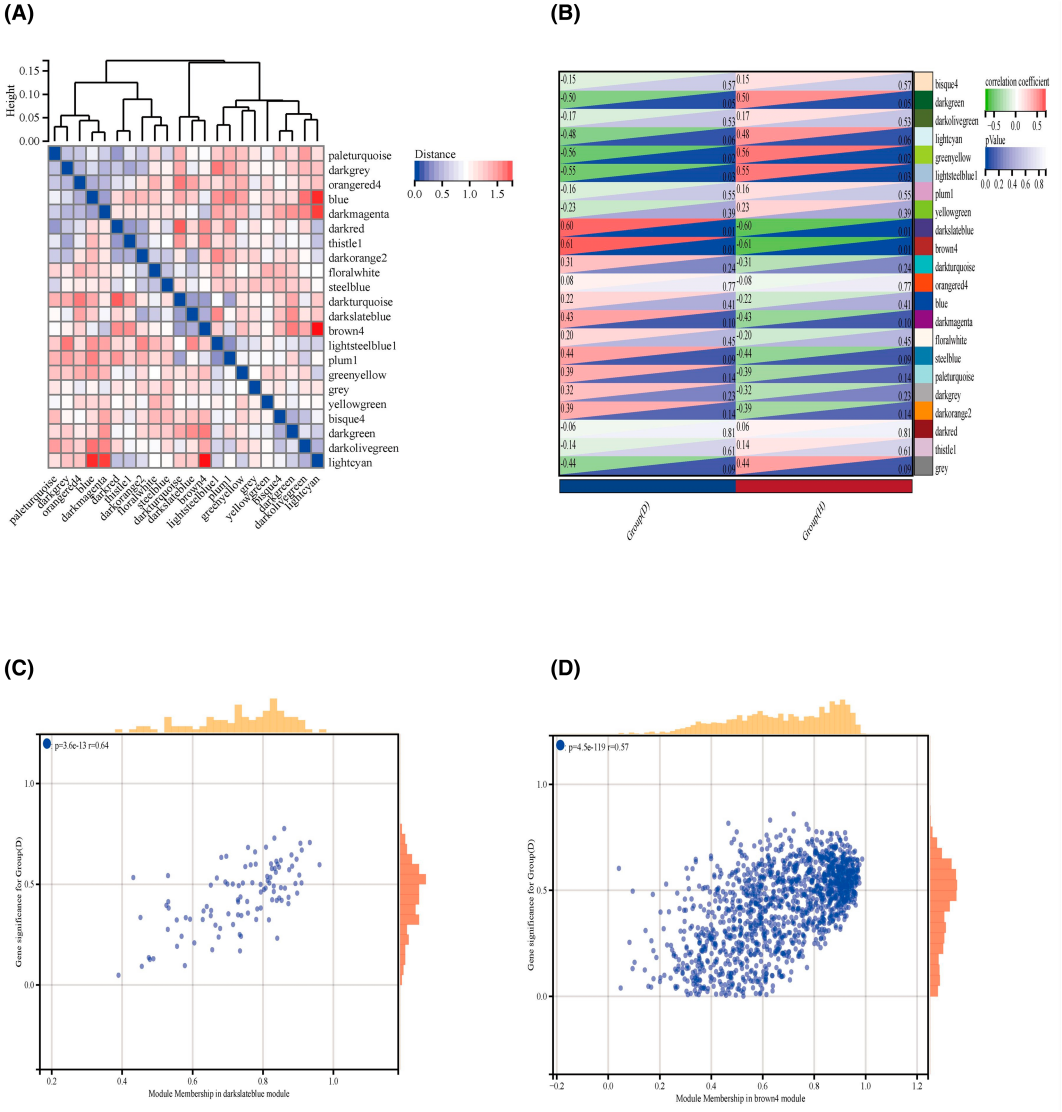
Weighted gene co‐expression network (WGCNA) analysis. (A) Heatmap of gene adjacency. (B) Heatmap of the association between modules and IVDD. The darkslateblue module and bown4 module are shown to be correlated significantly with IVDD. Numbers at the top and bottom brackets represent the correlation coefficient and p‐value. (C), (D) Correlation plot between module membership and gene significance of genes included in the darkslateblue module and bown4 module. H, normal control group; D, IVDD disease group.

The WGCNA approach was utilized in this study to determine the most relevant modules for IVDD. We chose a “soft” threshold of 9 (scale‐free *R*2 = 0.82) based on scale independence and average connectivity metrics when determining the optimal parameter values. The gene clustering dendrogram for the normal and IVDD groups is shown in Figure. We constructed 20 gene co‐expression modules (GCMs) based on this clustering dendrogram, with each module represented by a different hue (Figure 3A). Genes that could not be assigned to a module were assigned to the gray module and were omitted from further analysis.

In order to identify the modules with the highest correlation with IVDD, we calculated the correlation between IVDD and each GCM (Figure 3B). The results showed that two modules, darkslateblue, and brown4, had the highest correlation with IVDD (correlation coefficients of 0.61 and 0.60, respectively, *p* = 0.01). Therefore, we selected these two modules (containing a total of 523 genes) as the key modules for subsequent analysis.

Next, we calculated the module member associations between genes in the darkslateblue and brown4 modules and IVDD. The results showed a significant positive correlation between module membership and gene significance in both modules (Figure 3C, D, *r* = 0.64 and 0.57). Therefore, we can conclude that these two modules are significantly correlated with IVDD. The data in panels (C), (D) illustrate the correlation between module membership and gene significance for genes in the darkslateblue and brown4 modules. According to our analysis, the genes within these modules are involved in various biological processes related to IVDD. These processes include:

*Inflammatory response*: Genes such as CXCL1, IL1R1, IL1R2, and TLR4 are involved in the transmission and regulation of pro‐inflammatory signals, potentially playing a crucial role in the immune response during IVDD.

*ECM remodeling*: Genes like ACAN and MMP3 are closely related to the degradation and remodeling of the extracellular matrix, a key step in the pathogenesis of IVDD.

*Cell stress and apoptosis*: Genes such as SOD2, DAPK1, and BCL6 are associated with the cellular response to oxidative stress and the regulation of apoptosis, which are important in the survival and death of disc cells.

*Signal transduction and transcription regulation*: Genes like STAT3, FOS, and NF‐κB are involved in signaling pathways and transcription regulation, playing a significant role in modulating cellular behaviors and gene expression related to IVDD.

The diversity of these gene types reflects the complexity of IVDD, and the significant correlations in these modules further support their potential role as biomarkers or therapeutic targets in IVDD.

### 3.3 Hub gene screening and functional enrichment analysis

**FIGURE 4:**
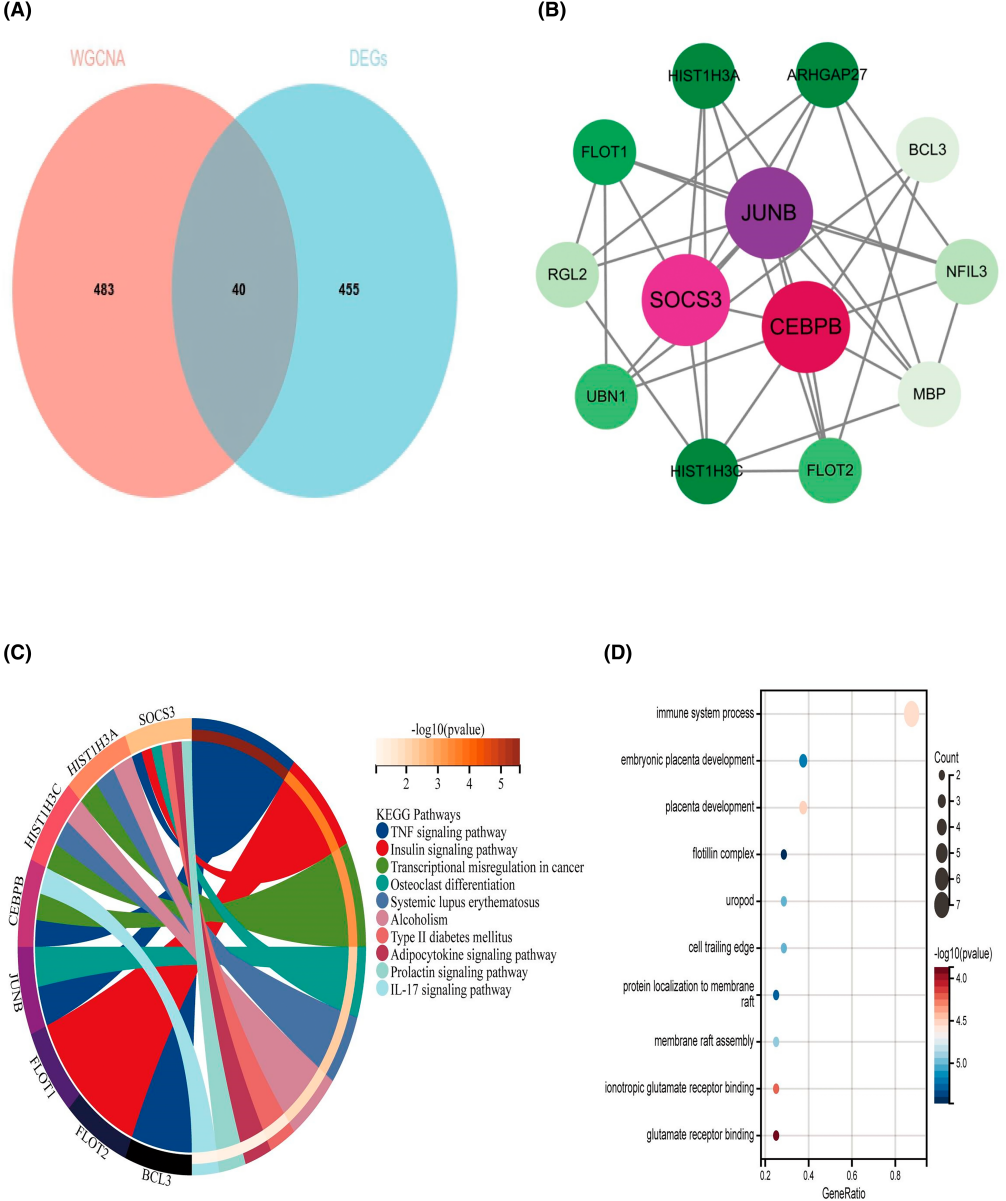
Hub gene screening and functional enrichment analysis. (A) Venn diagram showing the intersection between DEGs, and key modular genes obtained by WGCNA analysis. (B) Ten hub genes screened by the cytoHubba plugin, of which three red genes are core hub genes. (C) KEGG pathway analysis of 10 hub genes. (D) GO analysis of 10 pivotal genes for biological processes, cellular components, and molecular functions, respectively.

In order to construct a PPI network and screen out hub genes, we took the intersection of key module genes obtained from WGCNA analysis with DEGs to obtain 40 common genes (CGs) (Figure 4A) and then constructed a PPI network from the 40 CGs, which was inputted into cytoscape for visualization. We then analyzed these 40 genes using the DNNC algorithm in cytoHubba software to identify the top 10 hub genes and other important nodes (Figure 4B). To assess whether these 10 genes could reflect the pathogenesis of IVDD, we performed functional enrichment analysis. KEGG analysis showed that the 40 CGs were mainly enriched in signaling pathways such as “TNF signaling pathway” and “IL‐17 signaling pathway.” In addition, GO analysis showed that these CGs were mainly enriched in the areas of “intracellular signaling,” “immune response” and “positive regulation of NF‐kappaB transcription factor activity” (Figure 4C). “kappaB transcription factor activity” (Figure 4D). In summary, inflammatory and immune responses may be highly relevant to the pathogenesis of IVDD.

cytoHubba evaluates the importance of a node in the network by calculating its topological characteristics and centrality metrics, and the shading color of a node reflects its importance in the network. Typically, nodes with higher ratings or metric values are considered to have higher importance in the network. In order to identify these important nodes more clearly, we chose to use red panels to highlight them. The red panels indicate nodes that may have important functions or key roles in the network. Based on these considerations, we chose three red slab genes as hub genes, which are JUNB, SOCS3, and CEBPB.

### 3.4 Identification of hub genes and their clinical significance

**FIGURE 5:**
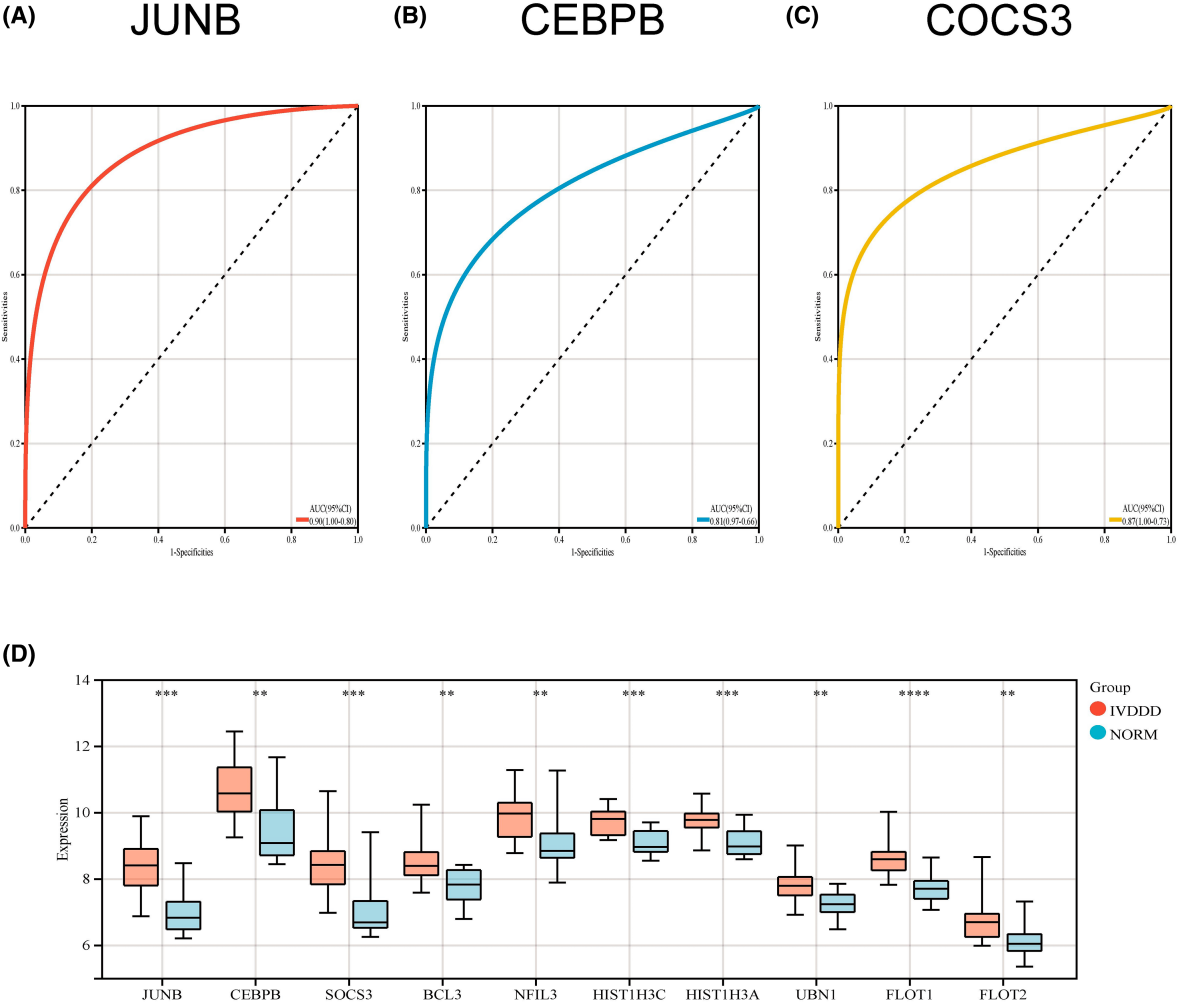
Identification and validation of hub genes. (A)–(C) The receiver operating characteristic curve analysis of hub genes(JUNB, CEBPB, and COCS3). (D) Histogram of overall expression of each pivotal gene in IVDD patients: Control samples in blue, disease samples in red, genes on the horizontal axis, gene expression levels on the vertical axis. *, *p* <0.05, **, *p* <0.01, ***, *p* <0.001.

To identify pivotal genes with high diagnostic value in IVDD, we performed a series of analyses. First, we assessed the diagnostic specificity and sensitivity of each candidate gene using ROC curves and calculated its AUC and 95% CI. The results showed that JUNB (AUC 0.90, CI 1.00–0.80), CEBPB (AUC 0.81, CI 0.97–0.66), and SOCS3 (AUC 0.87, CI 1.00–0.93) were of high value in the diagnosis of IVDD (Figure 5A–C).

Furthermore, in the GEO dataset after correcting for batch effects, we verified the differential expression of several key genes in normal and IVDD samples. The results showed that multiple genes, including JUNB, CEBPB, SOCS3, BCL3, NFIL3, HIST1H3C, HIST1H3A, UBN1, FLOT1, and FLOT2, were differentially expressed in IVDD samples compared to controls. Notably, these genes exhibited higher expression levels in IVDD samples, indicating their potential involvement in the pathogenesis of IVDD (Figure 5D).

### 3.5 Experimental validation results

**FIGURE 6:**
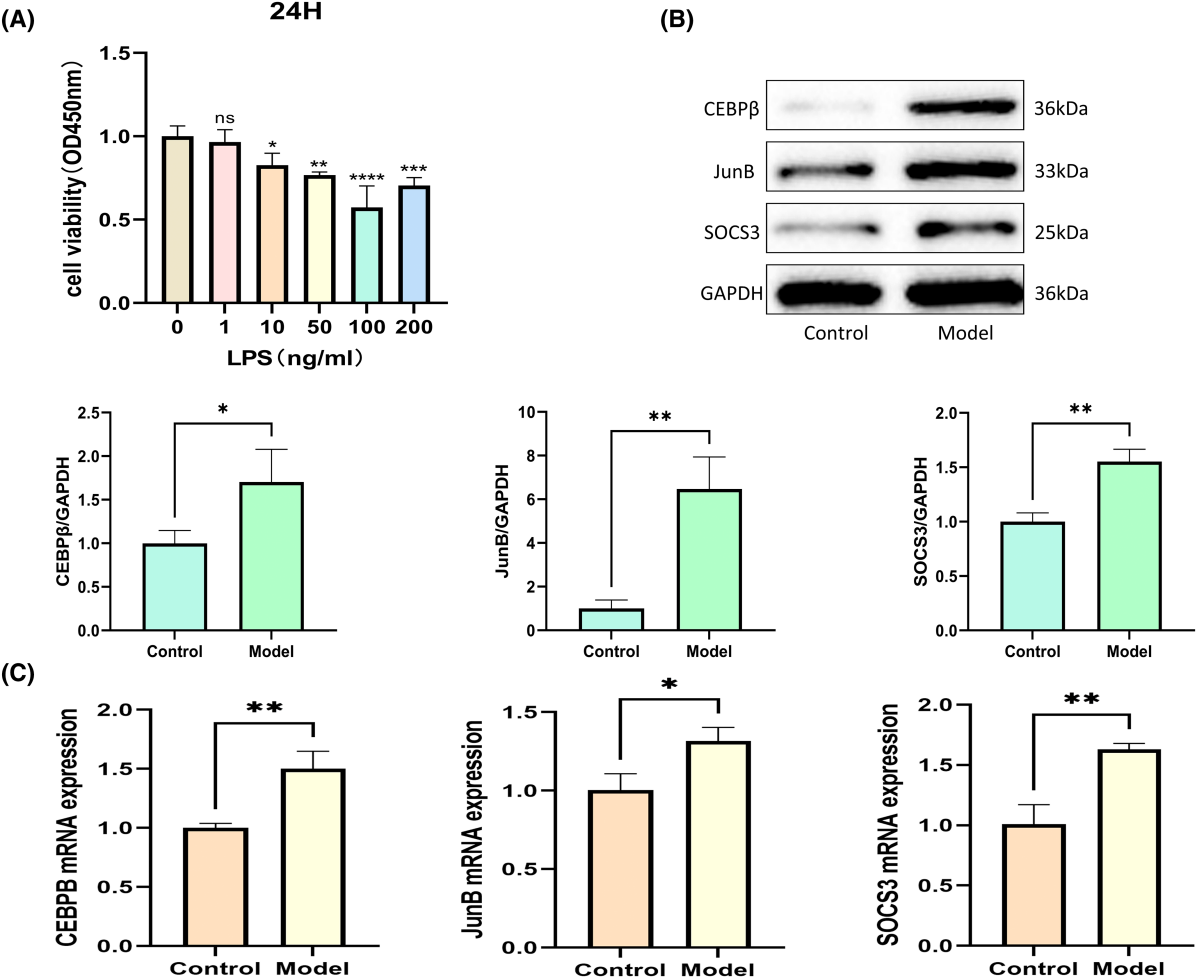
Experimental validation results. (A) The effect of different concentrations of LPS (0, 10, 20, 50, 100, and 200 ng/mL) on the viability of nucleus pulposus cells after 24 h of treatment. Cell viability was assessed using the CCK8 assay. *p <0.05 compared to the control group. (B) Expression levels of SOCS3, CEBPB, and JUNB proteins in nucleus pulposus cells treated with 0 or 100 ng/mL LPS for 24 h. GAPDH was used as a loading control. Representative blots are shown. (C) mRNA expression levels of SOCS3, CEBPB, and JUNB in nucleus pulposus cells treated with 0 or 100 ng/mL LPS for 24 h, measured by qRT‐PCR. The results represent the average of three independent experiments.

The CCK8 assay showed significant changes in NP cell viability with different LPS concentrations. Cell viability was lowest at the 100 ng/mL concentration (Figure 6A).

To further validate these results, we determined the expression levels of these three pivotal genes in normal NPCs and LPS‐induced IVDD cell models using WB and qRT‐PCR. WB analysis revealed that the expression of SOCS3, CEBPB, and JUNB proteins was significantly upregulated in the 100 ng/mL LPS‐treated group (Figure 6B). The qRT‐PCR results also showed that the mRNA levels of these genes were consistent with the protein levels (Figure 6C). Therefore, we can conclude that these three hub genes have strong potential as diagnostic biomarkers for IVDD. These genes may contribute to the early detection and diagnosis of IVDD by reflecting key molecular changes associated with the disease. Specifically, the identification and validation of these hub genes could lead to the development of diagnostic tools that improve the accuracy of IVDD diagnosis, potentially allowing for earlier and more targeted interventions. However, further studies, including clinical trials, are essential to determine the precise roles of these hub genes in the pathogenesis of IVDD and to rigorously evaluate their diagnostic accuracy, sensitivity, and specificity in clinical settings.

### 3.6 Prediction and validation of potential miRNAs targeting hub‐genes

**FIGURE 7:**
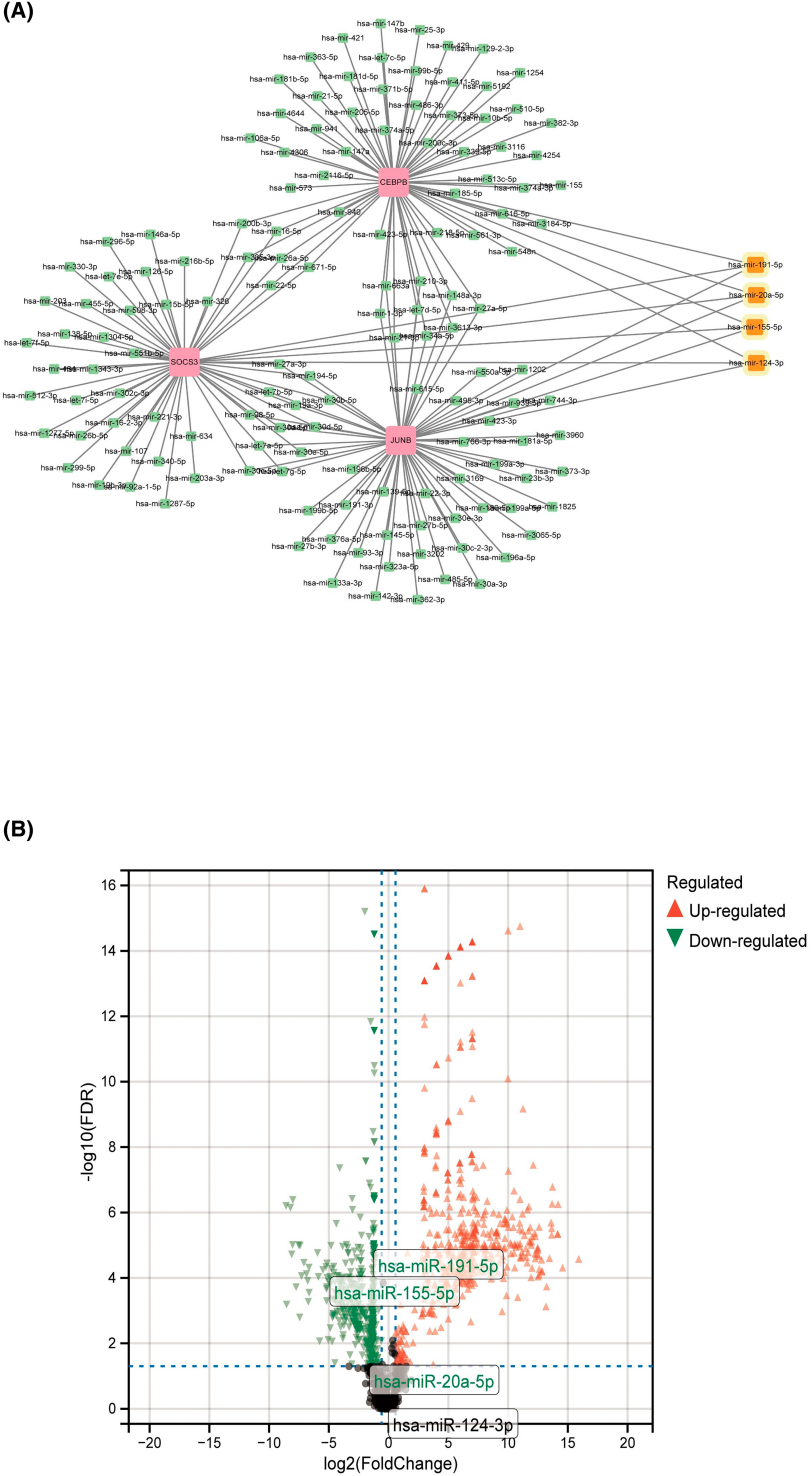
Screening of potential miRNAs targeting hub‐genes. (A) An Interaction network of three hub genes and potential miRNAs‐targeted. (B) The volcano plot of DE‐miRNAs between IVDD and normal control in GSE116726.

In this study, we utilized the miRNet database for target miRNA prediction of JUNB, CEBPB, and SOCS3 genes. Through the screening of this database, we predicted 145 potential miRNAs with the possibility of target regulation with these three hub genes. (Figure 7A) demonstrates the co‐regulation pattern of four miRNAs with these three hub genes. These four miRNAs were mir‐191‐5p, mir‐20a‐5p, mir‐155‐5p, and mir‐124‐3p. They played important roles in the regulation of transcription factors JUNB, CEBPB, and the repressor SOCS3, as identified through Limma differential analysis. It was found that mir‐191‐5p, mir‐20a‐5p, mir‐155‐5p, and mir‐125‐5p were the most effective in regulating these three hub genes. In IVDD samples, mir‐155‐5p showed significantly decreased expression, while mir‐124‐3p showed no significant difference (Figure 7B). Among the miRNAs analyzed, hsa‐let‐7d‐5p was found to be upregulated in IVDD samples. This particular miRNA has been identified as potentially involved in the regulation of the hub genes we discussed. The other miRNAs showed either decreased expression or no significant difference.

The prediction results of these target miRNAs provide important clues for further investigation of the regulatory networks of the three hub genes, JUNB, CEBPB, and SOCS3. Further functional experiments and validation will help to reveal the mechanism of action of these miRNAs in regulating gene expression and cell function and provide new targets and strategies for the treatment of related diseases.

## 4 DISCUSSION

IVDD is expected to become a prevalent orthopedic chronic disease due to the effects of an aging population and long office hours,^24^ and it is the leading cause of lower back pain,^25^ with studies suggesting that 80% of adults will be affected by low back pain at some point in their lives.^26^ IVDD is a change in the structure and function of the intervertebral disc and is commonly seen in pathological processes such as disc injury and age‐related degeneration. The development of disc degeneration is associated with several biological processes including apoptosis, inflammatory response, matrix degradation, and cytoskeletal reorganization.^27^, ^28^ As a key research area in orthopedic diseases, its treatment is limited and the fundamental reason is that the pathophysiologic mechanisms of IVDD are not fully understood.^29^ Bioinformatics research can help us better understand complex diseases and has identified a number of genes that play a key role in the pathogenesis of IVDD.^30^ In this study, we identified 495 DEGs and 2 important modules obtained by WGCNA analysis, established PPI network through the obtained intersections, and obtained Top10 hub genes by Cytoscape. After analyzing the obtained hub genes by GO analysis, we obtained that these CGs were mainly enriched in the fields of “intracellular signaling,” “immune response,” and “positive regulation of NF‐kappaB.” The KEGG analysis showed that these genes were mainly concentrated in the fields of “intracellular signaling,” “immune response,” and “positive regulation of NF‐kappaB transcription factor activity,” and the “TNF signaling pathway” and “IL‐17 signaling pathway.” “signaling pathway” and “IL‐17 signaling pathway.”

Previous studies have shown a close relationship between disc degeneration and the TNF signaling pathway, a pro‐inflammatory cytokine that plays an important role in disc degeneration. Studies have shown that activation of the TNF signaling pathway is involved in the onset and progression of disc degeneration. Activation of the TNF signaling pathway leads to the production of an inflammatory response, including the release of pro‐inflammatory cytokines and inflammatory mediators. These inflammatory mediators may cause destruction of disc tissue, promoting water loss and apoptosis. It was also found that activation of the TNF signaling pathway can induce the secretion of proteinases by disc cells, which may contribute to protein aggregation and tissue degradation, thus playing a role in the progression of disc degeneration. In addition, activation of the TNF signaling pathway can increase the sensitivity of inflammatory cells and neurons to painful stimuli, leading to pain associated with disc degeneration.^31^, ^32^, ^33^

There is a relationship between the IL‐17 signaling pathway and intervertebral disc degeneration. IL‐17 is a cytokine that, by binding to its receptor, activates a variety of inflammatory responses and is involved in the onset and progression of several inflammatory diseases. IVDD is a common intervertebral disc disease, and there is an inflammatory response involved in its onset and progression. Recent studies have shown that the IL‐17 signaling pathway may play a role in the onset and development of disc degeneration. Some studies have found that the level of IL‐17 is positively correlated with the degree of degeneration, and IL‐17 may promote the process of IVDD by regulating ECM degradation, inflammatory response, neoangiogenesis, cellular autophagy, and senescence, and inhibiting the IL‐17 signaling pathway can reduce the degree and symptoms of disc degeneration.^27^, ^34^

Through further screening, we selected JUNB, SOCS3, and CEBPB as the final hub genes. JUNB is a basic leucine zipper‐containing transcription factor belonging to the JUN family, including JUND and c‐JUN, which is involved in the regulation of gene expression and influences the biology of cell proliferation, differentiation, and apoptosis. JUNB is elevated in human chondrocytes and OA cartilage, as well as mouse OA JUNB is elevated in human chondrocytes and OA cartilage as well as mouse OA cartilage. In addition, upon activation of cytokines such as interleukin (IL)‐1β, JUNB can bind to the promoter of matrix metalloproteinase 13 (MMP13), promoting the expression of MMP13 and decreasing the expression of collagen II,^35^ which is similar to the mechanism that causes the degradation of extracellular matrix in NPCs in IVDD. Li et al. found that JUNB was overexpressed in damaged cartilage of knee OA patients and promoted osteoarthritic cartilage degeneration by inhibiting autophagy and anabolism and enhancing apoptosis and catabolism through direct targeting of the FBXO21 promoter.^36^ Due to the similarity between osteoarthritis and IVDD in physiopathological molecular mechanisms, biological processes such as inflammatory response, cell proliferation, and apoptosis play important roles in both diseases, JUNB gene may be involved in the process of disc degeneration, but the related mechanisms still need to be validated by further studies, and in‐depth studies can help to provide new directions and strategies for the treatment and prevention of IVDD.

Suppressor of cytokine signaling 3 (SOCS3) is a protein‐coding gene that is involved in the regulation of cell signaling pathways and negative feedback regulation. SOCS3, as an important negative regulator molecule, plays a key role in inflammatory response and immune regulation. It has been shown that SOCS3 can negatively regulate the signaling pathways of multiple inflammatory factors, including IL‐6, IL‐1β, and TNF‐α. When SOCS3 is highly expressed, it inhibits the signaling of inflammatory factors, resulting in ineffective negative regulation of inflammatory responses, thus promoting inflammatory responses and degeneration of intervertebral disc tissues. High expression of SOCS3 promotes the occurrence of apoptosis. In intervertebral disc degeneration, apoptosis is an important cell injury mechanism. When SOCS3 is highly expressed, it inhibits the negative regulatory pathway of apoptosis, leading to an increase in apoptosis and further accelerating the degeneration of intervertebral disc tissues. High expression of SOCS3 may also lead to abnormal proliferation and differentiation of intervertebral disc cells. It has been found that SOCS3 can inhibit the signaling pathways of various growth factors, such as EGF and FGF, thus affecting the proliferation and differentiation of intervertebral disc cells, and these abnormal proliferation and differentiation processes may lead to degeneration and structural damage of intervertebral disc tissues. In a recent study, Wang et al. suggested that SOCS3 may be a core gene involved in the development of IVDD and obtained that the expression of SOCS3 was higher in IVDD than in normal tissues by analysis of the GEO database, which is consistent with our validation results.^37^, ^38^, ^39^ However, the specific mechanism of action of SOCS3 involved in IVDD needs to be clarified by further studies.

CCAAT/enhancer binding protein beta (CEBPB) is an important regulator of the cell cycle and cell differentiation.^40^ Recent studies have found an association between CEBPB, a transcription factor that regulates the expression of a variety of genes, and IVDD. Zhang et al. found that CEBPB expression was elevated in human lumbar intervertebral joint degeneration. Further studies showed that CEBPB interacts with Runt‐related transcription factor 2 (RUNX2), a key bone and chondrocyte differentiation factor that plays an important role in skeletal tissues and that CEBPB and RUNX2 together promote chondrocyte apoptosis. In addition, researchers found that the expression levels of CEBPB and were positively correlated with the severity of IVDD, which implies that CEBPB may play an important role in the pathogenesis of IVDD.^41^ Zhou et al. found that the expression of CEBPB was significantly increased when TNF‐α and IL‐1β treated myeloid cells. Further studies showed that miR‐155 directly inhibited CEBPB expression by binding to the 3′ untranslated region of CEBPB. In addition, miR‐155 overexpression suppressed TNF‐α and IL‐1β‐induced transcription factor activity and production of inflammatory factors (e.g., IL‐6 and IL‐8). Further investigation of the interaction between miR‐155 and CEBPB may help us to better understand the pathogenesis of IVDD and provide new targets for the treatment of this disease.^42^

In addition, we predicted target‐regulated miRNAs for these three hub genes, namely mir‐191‐5p, mir‐20a‐5p, mir‐155‐5p, and mir‐124‐3p. Generally, primary miRNA is produced in the nucleus and further processed into mature miRNA in the cytoplasm by Dicer enzymes. The addition of RNA‐inducible silencing complexes can target and inhibit the translation of a wide range of mRNAs, thereby regulating approximately 30% of human protein‐coding genes and a variety of intracellular processes including cell proliferation, apoptosis, and cytokine release.^42^ miRNA dysregulation is associated with a variety of pathological conditions, including cancer and cardiovascular disease, as well as osteoarthritis and IVDD.^43^, ^44^, ^45^, ^46^ This has generated interest in miRNAs, their role in disease progression, and their potential as novel biomarkers and therapeutics. Among the miRNAs analyzed, hsa‐let‐7d‐5p was found to be upregulated in IVDD samples. This particular miRNA has been identified as potentially involved in the regulation of the hub genes we discussed. The other miRNAs showed either decreased expression or no significant difference.

miR‐155‐5p may play an important regulatory role in IVDD. Srikanth N. MD et al. found that the expression level of mir‐155‐5p was significantly reduced in patients with disc degeneration. In addition, it was found that the reduction of mir‐155‐5p was negatively correlated with the severity of intervertebral disc pain and the degree of degeneration. mir‐155‐5p, as a microRNA, plays an important regulatory role within the cell. Further studies revealed that mir‐155‐5p inhibited apoptosis and inflammatory responses in NPCs and promoted collagen synthesis. Therefore, a decrease in mir‐155‐5p may lead to an increase in apoptosis of NPCs, an increase in inflammatory response, and a decrease in collagen synthesis, leading to the onset and progression of intervertebral disc degeneration. In addition, the authors found that mir‐155‐5p can influence the development of disc degeneration by regulating the expression of its downstream molecules. For example, it was found that a decrease in mir‐155‐5p can lead to overexpression of inflammatory mediators, which promotes the progression of disc degeneration.^47^ In summary, the reduction of mir‐155‐5p may be involved in the process of disc degeneration, which is consistent with our findings (Figure 7B). Thus mir‐155‐5p has the potential to be a new target in the diagnosis and treatment of disc degeneration. The role of mir‐191‐5p, mir‐20a‐5p, and mir‐124‐3p in IVDD and how to regulate the three pivotal genes is still limited and needs to be further explored.

However, there are still some limitations in our study. in the WGCNA analysis, we only investigated two key module‐related genes, which may have overlooked others. Although we combined three datasets, the sample size is still limited, and more clinical samples as well as further experiments will be needed subsequently to deeply investigate the results we obtained. Although this study used datasets from a variety of sources, these represent the diversity of IVDD. However, we recognize that the sample size is limited and the heterogeneity of the data may affect the reliability of the results. Therefore, we plan to collect more samples and validate them using more stringent criteria in future studies.

One significant limitation is the validation process, which was conducted using only a single immortalized cell line. Although this approach provided valuable insights, it may not fully capture the complexity of IVDD in vivo. The use of immortalized cell lines, while convenient for preliminary analysis, may limit the generalizability and translatability of our findings to clinical settings. Future research should aim to validate these findings using primary cell cultures or tissue samples from patients with IVDD to enhance the relevance and applicability of the results. Additionally, in vivo studies and the use of multiple cell lines or patient‐derived models could further substantiate the biomarkers identified and provide a more comprehensive understanding of their roles in IVDD.

## 5 CONCLUSION

Synthesizing our findings, we successfully identified key biomarkers and related mechanisms associated with IVDD. By analyzing gene expression profiles and constructing PPI networks, we obtained 10 hub genes, among which JUNB, SOCS3, and CEBPB showed a trend of overexpression as core genes in the IVDD model group. Functional enrichment analysis also revealed that these genes were enriched in important pathways such as TNF signaling pathway and IL‐17 signaling pathway.

In addition, we found that mir‐191‐5p, mir‐20a‐5p, and mir‐155‐5p showed decreased expression in IVDD samples by predicting co‐regulated miRNAs, while mir‐124‐3p showed no significant difference. The involvement of these miRNAs may play a regulatory role for regulating the expression of core genes in the pathogenesis of IVDD.

Taken together, our findings provide insights into the pathogenesis of IVDD. the three core genes, JUNB, SOCS3, and CEBPB, as well as their regulated miRNAs (mir‐191‐5p, mir‐20a‐5p, and mir‐155‐5p), may play important regulatory roles in the development of IVDD. These findings contribute to our better understanding of the pathophysiologic process of IVDD and provide new targets and strategies for future clinical diagnosis and treatment.

## AUTHOR CONTRIBUTIONS

*Conceptualization*: ZL. *Data curation*: XX. *Formal analysis*: SG, KC. *Methodology*: WC, DG. *Visualization*: TL, JL. *Writing—original draft*: DZ. *Review and editing*: YM.

## FUNDING INFORMATION

The present study was supported in part by research grants from the Project Supported by the Sichuan Science and Technology Department Project Development Project (nos. 2022YFS0391), the Program for Special Project of Traditional Chinese Medicine scientific research of Sichuan Science and Traditional Chinese Medicine Administration (nos. 2020LC0228), and the Program for Luzhou Municipal People's Government—Southwest Medical University science and technology strategic cooperation climbing project (nos. 2021LZXNYD‐D02). And Luzhou City science and technology research and development projects (2022‐SYF‐42). And Sichuan Provincial Science and Technology Plan Joint Innovation Special Project (2022YFS0609‐B3). And Luzhou city science and technology innovation seedling cultivation plan (2022‐RCM‐178).

## CONFLICT OF INTEREST STATEMENT

The authors declare that they have no known competing financial interests or personal relationships.

## Supporting information


Data S1

Data S2

Data S3

Data S4

## Data Availability

The datasets used and/or analyzed during the current study available from the corresponding author on reasonable request.
