# Supplementary material for: Identifying critical modules and biomarkers of intervertebral disc degeneration by using weighted gene co‐expression network
Source: JOR Spine. 2024 Oct 18;7(4):e70004. doi: 10.1002/jsp2.70004 (PMC11487274; doi:10.1002/jsp2.70004)
Supplement: Supplementary file 4 — Data S4 [file JSP2-7-e70004-s001.docx]

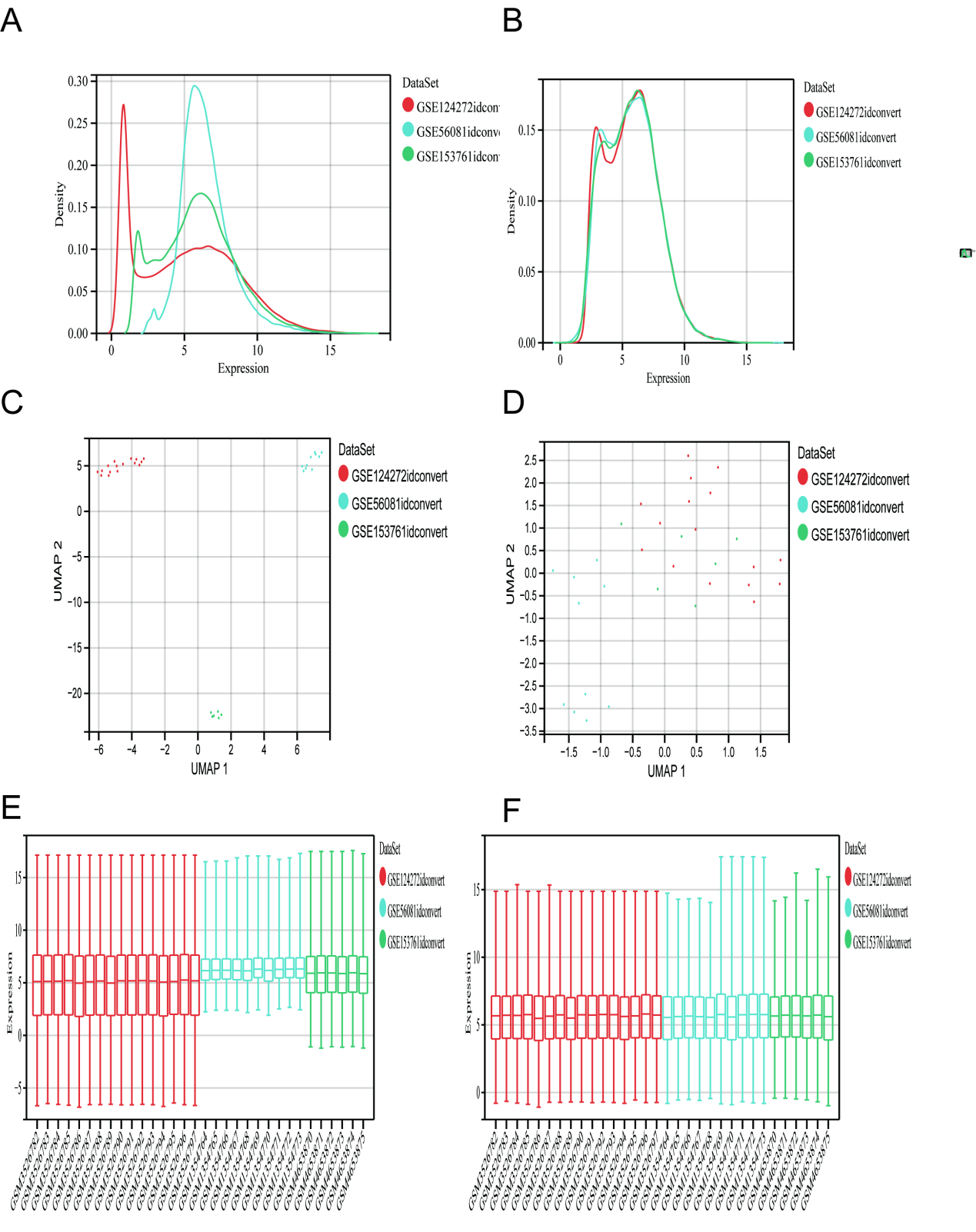


**GEO data de-batching.** (A) From the density plot, we can observe that the sample distribution of each dataset before removing the batch effect varies greatly, suggesting a batch effect. (B) After removing the batch effect, the distribution of data between the datasets tends to be consistent, with similar means and variances. (C) Principal Component Analysis (PCA) between datasets before de-batching. (D) Principal Component Analysis (PCA) between datasets after de-batching. (E) Statistical data on gene expression levels in the dataset before de-batching. (F) Statistical data on gene expression levels in the integrated dataset after de-batching.


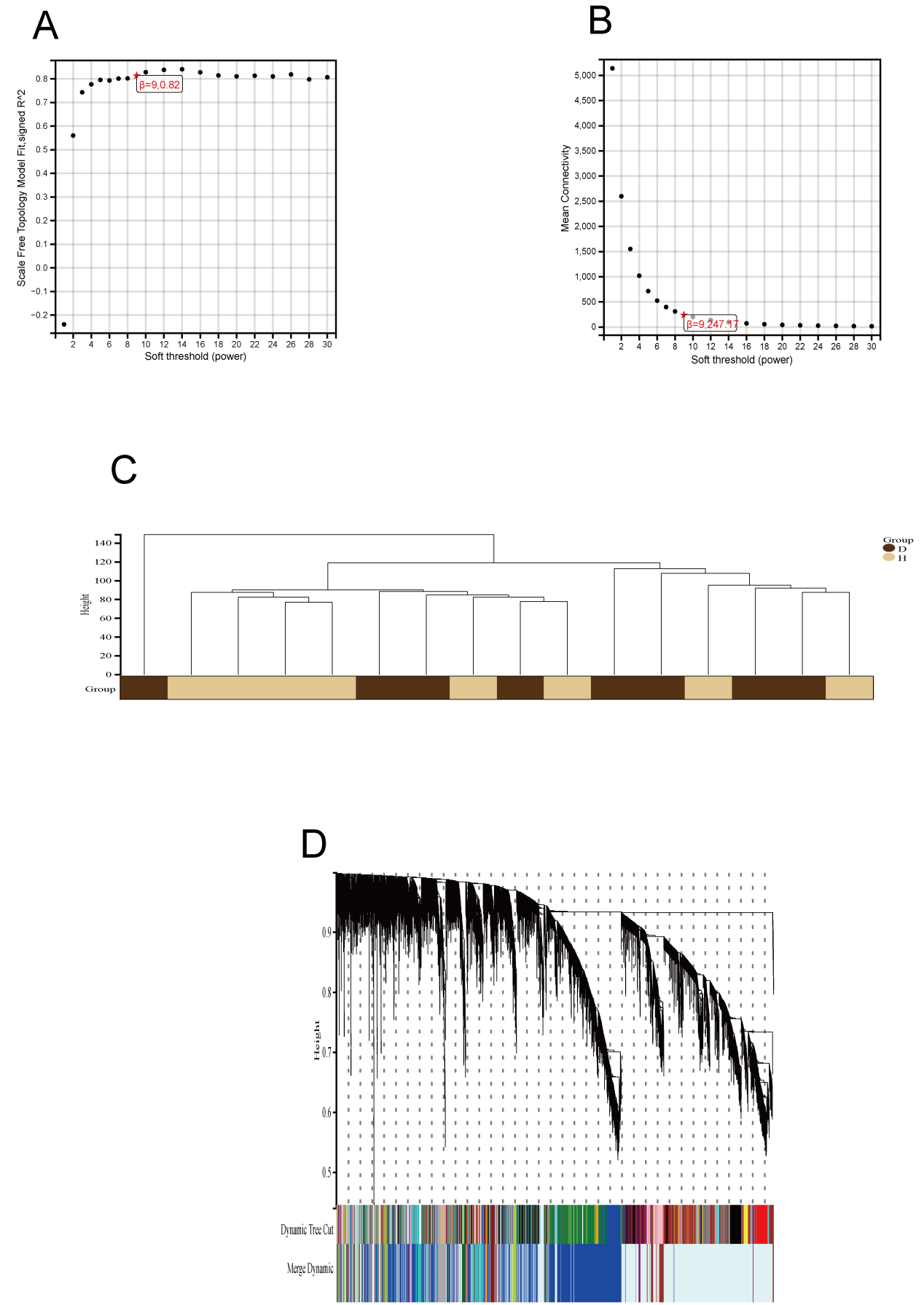


**Weighted gene co-expression network (WGCNA) analysis.** (A, B) Screening for soft thresholds based on scale independence and average connectivity. (C) Clustering dendrogram of the IVDD and controlsamples. (D) Gene co-expression modules indicated by different colors under the gene tree.


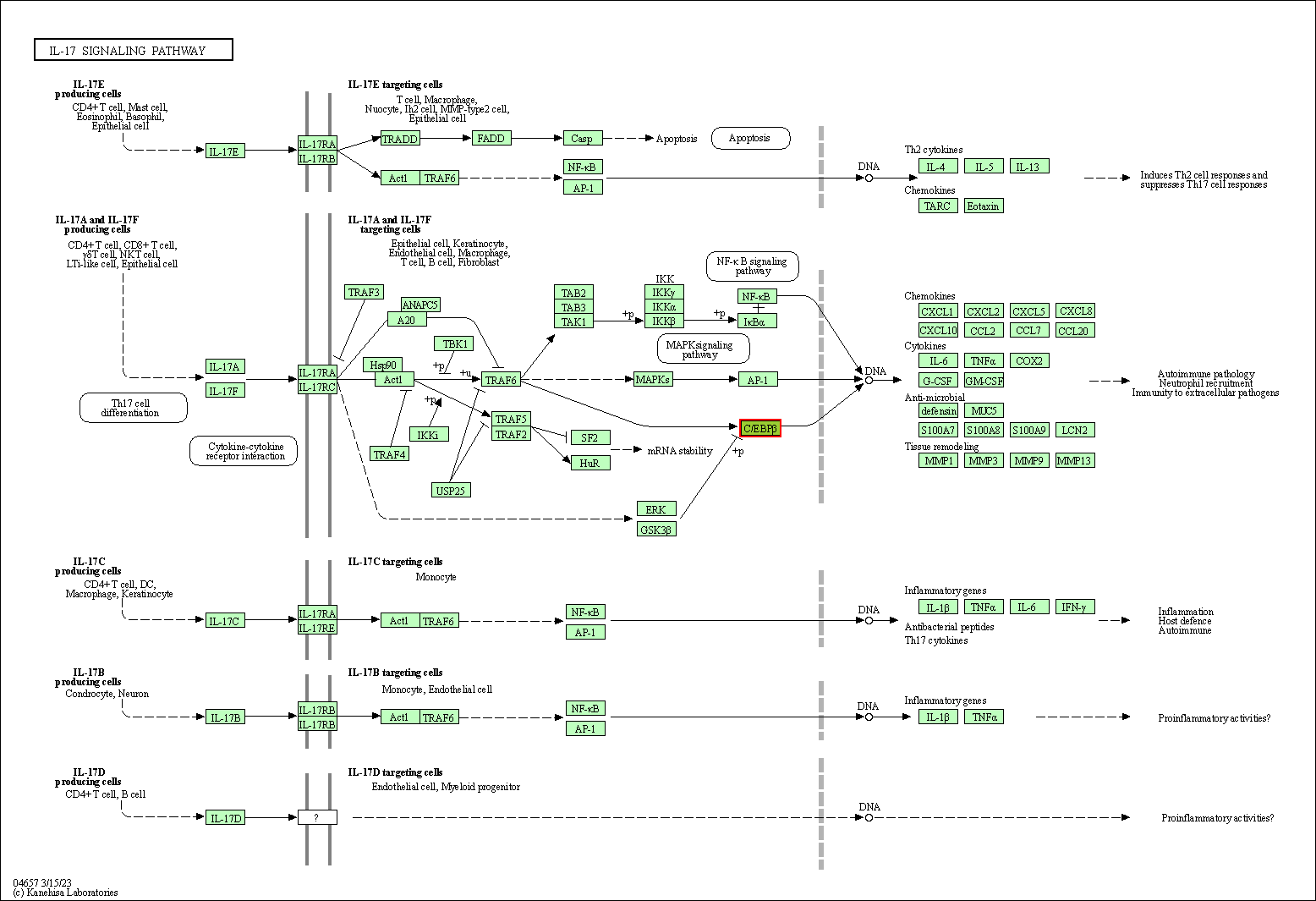


**Critical gene in IL-17 signalling pathway.**

**
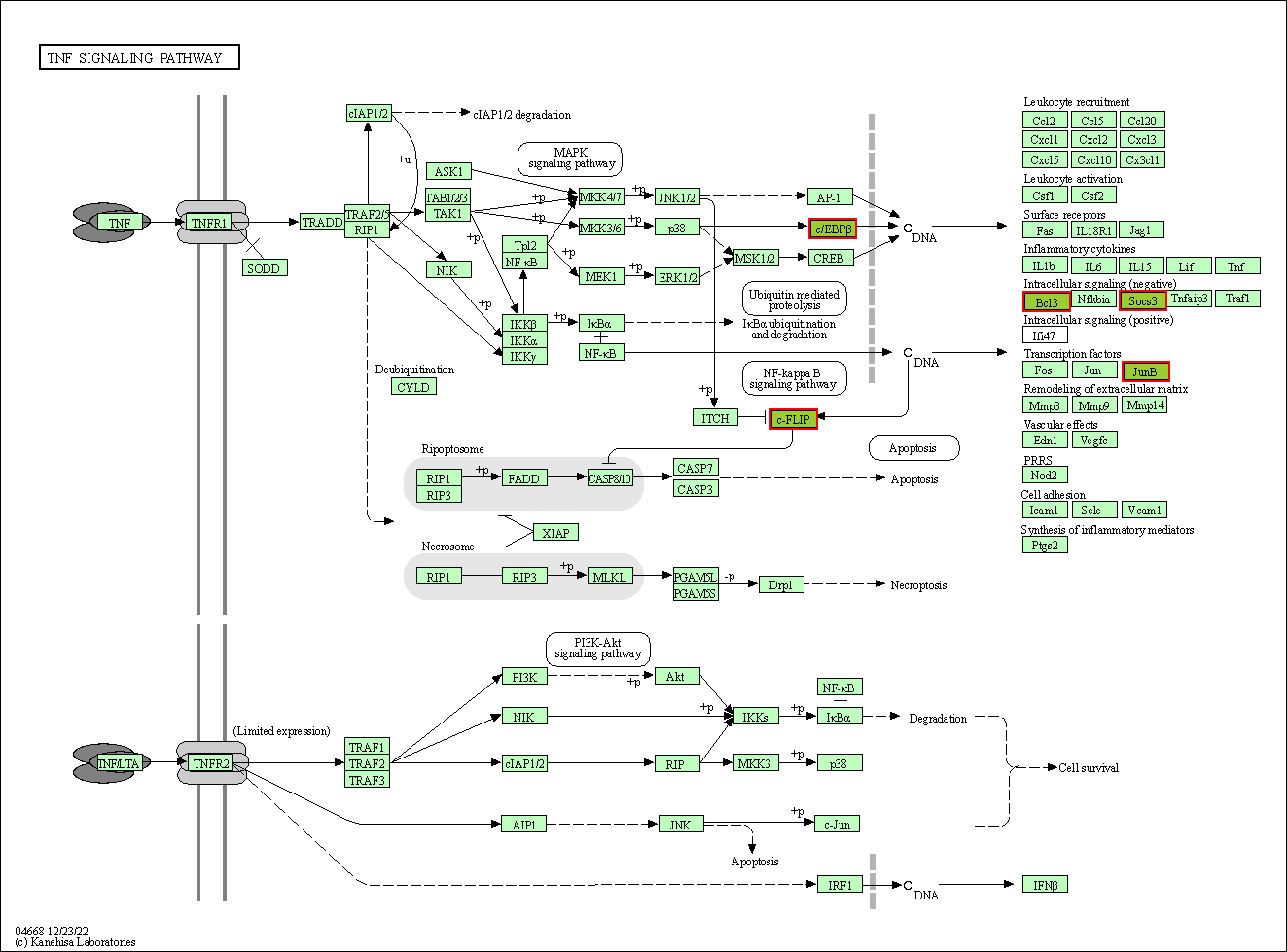
**

**Critical gene in TNF signalling pathway.**
